# In Vivo Comparison of Chlorine-Based Antiseptics versus Alcohol Antiseptic for Surgical Hand Antisepsis

**DOI:** 10.1155/2020/3123084

**Published:** 2020-09-24

**Authors:** Zhannur Myltykbayeva, Galina Kovaleva, Azamat Mukhitdinov, Sandugash Omarova, Rashid Nadirov

**Affiliations:** ^1^Al-Farabi Kazakh National University, Almaty 050040, Kazakhstan; ^2^M. Aikimbayev Kazakh Scientific Center for Quarantine and Zoonotic Diseases, Almaty 050040, Kazakhstan; ^3^Abai Kazakh National Pedagogical University, Almaty 050010, Kazakhstan

## Abstract

Despite being commonly used as effective preparation for surgical hand antisepsis, alcohol solutions have major drawbacks, such as drying effect, emergence of hand eczema, and other diseases. This study aimed to demonstrate the effectiveness of sodium hypochlorite (NaOCl) and hydrogen peroxide (H_2_O_2_) as antiseptic in comparison to single sodium hypochlorite and 70% ethanol. In 5-day tests, the effects of 3 antiseptics were established according to standard test methods. The antiseptics were applied to the hands of 82 volunteers, and samples of bacteria were collected on days 1 and 5, immediately after drying and 6 hours later after antiseptic application. Student's *t* test and ANOVA were applied in a statistical study. The NaOCl with H_2_O_2_ composition demonstrated noninferiority to both sodium hypochlorite only and alcohol products and superiority to these antiseptics on day 5 (*P* < 0.05 at a significance level of 5% for each comparative trial in this day) at equivalence margin of 20%. The effectiveness of the NaOCl plus H_2_O_2_ composition as an antiseptic was explained by the formation of singlet oxygen in the system. Together, these data suggest that NaOCl and H_2_O_2_ may be an effective hand antisepsis that avoids the drawbacks seen with alcohol solutions.

## 1. Introduction

During surgery, multiresistant bacterial flora of the hands of a surgical team is a potential source of pathogens that can be considered as a cause of surgical site infection (SSI). Skin antiseptics have been proven to rapidly and significantly reduce bacteria counts on the surgeons' hands [1]. Among the most commonly used antiseptics for these purposes are alcohol-based ones, which are very effective in preventing infections in healthcare centres [2, 3]. However, being recognized as an active agent to prevent SSI in developed countries, alcohol-based antiseptics have the following serious limitations: (i) drying effect on the hands [4]; (ii) chronic hand eczema caused by alcohol-based antiseptics [5, 6]; (iii) liver cirrhosis, fetal alcohol syndrome and cancer caused by ingestion of alcohol [7]. Further, alcohol-based antiseptics are expensive [8] and must be stored away from flame due to high flammability [9].

Hypochlorous acid (HOCl) is an acceptable alternative to alcohol in terms of being explored as antiseptic and is effective against a wide range of microorganisms [10–12]. HOCl is produced by white blood cells, making it an endogenous substance that can be tolerated in low concentrations by humans [13]. Not only HOCl but also its salts can act as antiseptic; it was reported in [14] that 0.05% of sodium hypochlorite (NaOCl) can also be used as an antiseptic.

As demonstrated by Petrosyan [15], a solution of 0.2–0.3% NaOCl in combination with 3% hydrogen peroxide (vol. 9 : 1) buffered by monosodium phosphate (NaH_2_PO_4_) is an effective antiseptic for the treatment of the hands of surgeons and medical staff. The specified composition is very easy to be prepared for direct use by mixing the finished components (sodium hypochlorite, water, hydrogen peroxide, and monosodium phosphate). Further, it is possible to prepare a solution by electrolysis of available sodium chloride [10].

However, we are not aware of any published investigations evaluating this chlorine-based antiseptic in comparison with alcohol-based ones, following reputable test standards. In the present work, we compared the effectiveness of the indicated antiseptic with ethanol (70%) as well as with sodium hypochlorite solution. The study was designed as a noninferiority investigation. The ASTM E1115, Standard Test Method for Evaluation of Surgical Hand Scrub Formulation [16], and the in vivo method specified by the US Food and Drug Administration for evaluation of surgical hand scrubs were used for comparison of the specified composition and substances as antiseptics [17]. In vivo methods including hands testing for levels of skin microbiota before and after antisepsis are comparatively easy to perform; however, it is subject to many uncontrollable factors [18, 19]. Nevertheless, such methods are widely used to test the capacity of a formulation to reduce the level of transient microflora on the hands without regards to the resident flora.

## 2. Materials and Methods

### 2.1. Test Products

One combination (NaOCl plus H_2_O plus NaH_2_PO_4_) and two substances (NaOCl and C_2_H_5_OH) were compared in terms of antimicrobial activity.

The composition was prepared from commercial sodium hypochlorite (CAS No. 7681-52-9, Hebei Qige Biotechnology Co., Ltd.), monosodium phosphate (≥99.0%, Sigma-Aldrich), and double-distilled water. The 0.25 wt% of NaOCl solution was prepared and 4 g of NaH_2_PO_4_ was added to 1 L of the solution. The resulting solution (further designated as **Antiseptic I**) was used for evaluation. The fresh solution was prepared every day to ensure the stability of the composition.

The 0.25 wt% of NaOCl solution and the 70% ethanol solution (Sigma-Aldrich) are further designated as **Antiseptics II** and **III**, accordingly.

### 2.2. Participants

A total of 89 volunteers were recruited for the study.

Practice nurses were responsible for recruiting participants. Nurses provided an information sheet to each potential participant, and with agreement with terms and conditions specified, participants recorded their written informed consent. All the volunteers had to meet the inclusion criteria: free of any underlying chronic diseases, at least 18 years of age, not pregnant, and had no dermatologic conditions or injuries on the hands. Candidates having taking antibiotics or with a history of sensitivity to alcohol, latex, and detergents were excluded from the number of potential participants.

### 2.3. Pretreatment Phase

According to the informed consent, all the participants agreed not to expose their unprotected hands or forearms to any chemicals throughout one week before sample collecting. During the 2 hours before any sample collecting, participants refrained from washing their hands. At the end of the 1 week, baseline samples were collected from both hands of the participants, by using the glove juice technique [16], on Monday, Wednesday, and Friday.

Before sample collection, all the participants washed their hands and lower two-thirds of the forearms by using nonmedicated soap. Hands were exposed under running tap water for 30 seconds. For all the participants, average baseline counts were >5 log_10_ colony-forming units per hand.

### 2.4. Treatment Procedure

All participants were randomly divided into three groups, and each group was assigned to apply one of the test antiseptics (I, II, or III).

Before application of a test product, participants clipped fingernails to ∼1 mm free edge if necessary, removed all jewelry from hands and arms, and cleaned under fingernails with a nail cleaner.

During the 5 days, antiseptics were applied via the following regimen: once on days 1 and 5 and three times on days 2, 3, and 4. Samples were collected 4 times, on days 1 and 5: immediately after the antiseptic had air-dried and between 6 and 6.5 hours after drying.

### 2.5. Counting the Number of Bacteria

Powder-free, sterile latex gloves were placed on both of the participant's hands. Within 1 minute of donning the gloves, 75 ml of sterile stripping fluid was instilled into the glove. The wrist was secured, and an attendant massaged the hand through the glove in a standardized manner for 1 minute ± 5 seconds. A 5 ml aliquot of the glove juice was removed and diluted in 5 ml of Butterfield's phosphate buffer solution with product neutralizers (10^0^ dilutions). The 10^0^ dilution was then serially diluted in Butterfield's phosphate buffer solution with product neutralizers.

Duplicate spread plates were prepared from each of these dilutions on tryptic soy agar with 0.07% lecithin and 0.5% Tween 80 and incubated at 30°C ± 2°C for 48–72 h. Bacteria were counted by using the standard plate-counting procedure. Raw data (colony-forming unit (CFU)/mL) were converted to log_10_ CFU/hand.

Butterfield's phosphate-buffered solution and plating medium were evaluated following ASTM E-1054 [20] to ensure that the active components of the test products were effectively neutralized.

### 2.6. Statistics

Descriptive statistics were calculated using *Minitab* Statistical software. Comparison of the effectiveness of antiseptics was carried out following the ±20 rule [21]; antiseptics II and III were the comparators. A single trial for comparison of the antiseptic I versus antiseptics II and III was performed.

## 3. Results and Discussion

### 3.1. Participants Involved in the Study

**Figure 1:**
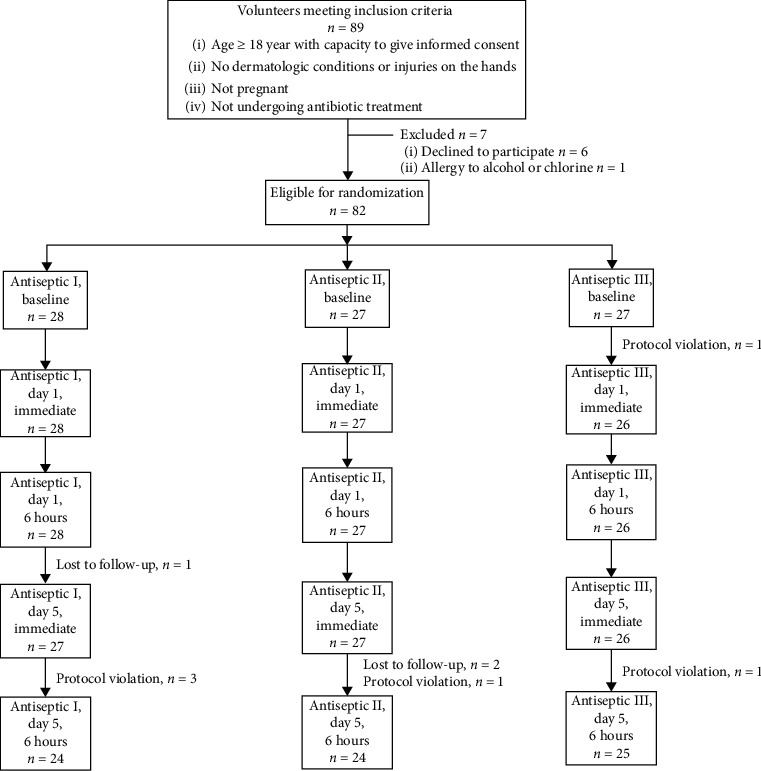
The pattern of enrollment, assignment, follow-up, and analysis of the study participants.

89 volunteers met inclusion criteria, 6 of whom declined to participate, and 1 was allergic to alcohol; thus, 82 participants were randomly divided into 3 groups for testing procedures. Antiseptics I, II, and III were applied to 28, 27, and 27 subjects, accordingly. Because of protocol violation as well as losing to follow-up by 8 participants during the 5-days testing period, only 74 participants were included in the analysis for the primary outcome (Figure 1).

The data about 82 subjects who started participating is given in Table 1.

### 3.2. Bacteria Count

The values of mean, standard deviation (SD), and standard error of the mean (SEM), calculated on the log_10_ microbial recoveries, are presented in Table 2. The log_10_ values for samples collected in each of 3 days on baseline week were averaged before including in the Table.

Analysis of variance (ANOVA) used for comparing 3 baseline means; the *P* value was calculated to be 0.375, indicating that baselines did not significantly differ from each other.

### 3.3. Noninferiority and Superiority Trials

The log_10_ reduction from the baseline at each point specified in Table 1 compared between the Antiseptic I and Antiseptic II, as well as between the antiseptic I and antiseptic III, by using the Student's *t* test.

To determine whether the **Antiseptic I** is no less effective than **antiseptics II** and **III**, a confidence interval (CI) approach was used. A two-sided 95% confidence interval was applied for the noninferiority test as this approach preserves consistency between significance testing and subsequent estimation [22].

**Figure 2:**
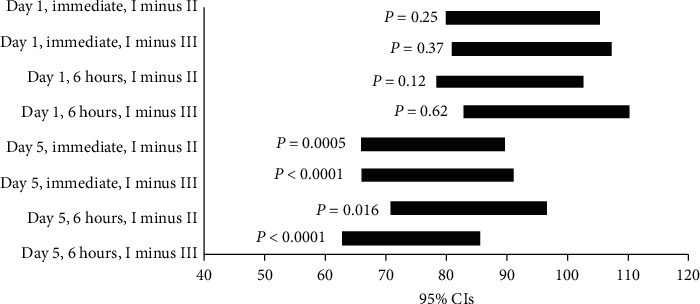
95% CIs for percent differences.

Figure 2 demonstrates a graphical representation for the noninferiority test.

It is indicated that the CIs for all comparisons (**Antiseptic I** minus **Antiseptic II** and **Antiseptic I** minus **Antiseptic III** for all 8 cases) lie above zero. *P* values were calculated for all cases as seen in Figure 2. As shown, **Antiseptic I** is noninferior to both **Antiseptics II** and **III** and in fact demonstrated superiority to both reference antiseptics in day 5 (*P* < 0.05 for each comparative trial in this day).

### 3.4. Adverse Reactions

No adverse reactions attributable to the antiseptic application were detected.

### 3.5. Possible Reason for Antiseptic NaOCl + H_2_O_2_ + NaH_2_PO_4_ Effectiveness

The combined presence of sodium hypochlorite and hydrogen peroxide in the solution leads to the formation of free radicals and/or active oxygen [23]. The possibility of the formation of singlet oxygen by the following reactions was shown in [24]:

(1) $$\begin{array}{c} {\text{OCl}}^{-}{+\text{ H}}^{+}\;⟶\text{ HOCl} \end{array}$$

(2) HOCl+ H2O2⟶H++ Cl−+ H2O+ O2Δ1↑

Singlet oxygen is a strong bactericidal agent and acts as an effective antiseptic against multiple pathogens, including microbes, virus, and also cancer cells. Besides, singlet oxygen has a high-penetrating ability into the deeper layers of the skin [15, 25]. The presence of monosodium phosphate is explained by its role in stabilizing the pH of the mixture.

## 4. Conclusions

In this study, we found that the mixture of NaOCl and H_2_O_2_ (and also NaH_2_PO_4_ as a buffering component) demonstrated noninferiority and on day 5 superiority toward 70% ethanol solution (and also toward single NaOCl solution) at the statistical significance of 5% level. Together, this study presents evidence that an antiseptic consisting of NaOCl and H_2_O_2_ may be a promising alternative to traditional alcohol hand antiseptics.

## Figures and Tables

**Table 1:** Demographic data on study participants.

Sex	Age group	Number of participants for each testing antiseptics		
		I	II	III
Male	18–27	3	2	—
	28–37	—	5	3^4^
	38–47	10^1^	2	8
	48–57	—	7^3^	3

Female	18–27	—	—	1
	28–37	—	1	—
	38–47	8^2^	7	8
	48–57	7	3	4^5^

The number of participants excluded from the primary outcome analysis: ^1, 4, 5^–1; ^2, 3^–3.

**Table 2:** Statistics of log recoveries in log_10_ CFU/hand for testing antiseptics in different times.

Testing	Antiseptic	Number of participants	Mean	Standard deviation	Minimum	Maximum	Standard error
Baseline	I	56	6.06	0.58	5.02	6.93	0.08
	II	54	5.91	0.62	5.04	6.98	0.08
	III	54	5.95	0.56	5.07	6.92	0.08

Day 1, immediate	I	28	3.71	0.92	2.31	5.21	0.17
	II	27	4.00	0.94	2.21	6.14	0.18
	III	26	3.94	0.95	2.42	5.64	0.19

Day 1, 6 hour	I	28	3.68	0.96	2.03	5.41	0.18
	II	27	4.06	0.85	2.32	5.67	0.16
	III	26	3.81	0.95	2.38	5.99	0.19

Day 5, immediate	I	27	3.24	0.92	2.10	5.32	0.18
	II	27	4.16	0.89	2.88	5.83	0.17
	III	26	4.42	0.93	3.06	5.92	0.18

Day 5, 6 hour	I	24	3.46	0.91	2.07	5.57	0.19
	II	25	4.13	0.94	2.68	5.64	0.19
	III	25	4.66	0.94	2.71	5.99	0.19

## Data Availability

The data supporting the results can be made available from the corresponding author upon request.

## References

[1] Maier A., Ovesen J. L., Allen C. L. (2015). Safety assessment for ethanol-based topical antiseptic use by health care workers: evaluation of developmental toxicity potential. *Regulatory Toxicology and Pharmacology*.

[2] Kanwar A., Kumar J. A., Ng-Wong Y. K. (2019). Evaluation of an alcohol-based antiseptic for nasal decolonization of methicillin-resistant *Staphylococcus aureus* in colonized patients. *Infection Control & Hospital Epidemiology*.

[3] Malani A., Trimble K., Parekh V., Chenoweth C., Kaufman S., Saint S. (2007). Review of clinical trials of skin antiseptic agents used to reduce blood culture contamination. *Infection Control & Hospital Epidemiology*.

[4] Czerwinski S. E., Cozean J., Cozean C. (2014). Novel water-based antiseptic lotion demonstrates rapid, broad-spectrum kill compared with alcohol antiseptic. *Journal of Infection and Public Health*.

[5] Cimiotti J. P., Marmur E. S., Nesin M., Hamlin-Cook P., Larson E. L. (2003). Adverse reactions associated with an alcohol-based hand antiseptic among nurses in a neonatal intensive care unit. *American Journal of Infection Control*.

[6] Kampf G., Löffler H. (2003). Dermatological aspects of a successful introduction and continuation of alcohol-based hand rubs for hygienic hand disinfection. *Journal of Hospital Infection*.

[7] Bessonneau V., Clément M., Thomas O. (2010). Can intensive use of alcohol-based hand rubs lead to passive alcoholization?. *International Journal of Environmental Research and Public Health*.

[8] Boyce J. (2001). Antiseptic technology: access, affordability, and acceptance. *Emerging Infectious Diseases*.

[9] Wolfe M. K., Gallandat K., Daniels K., Desmarais A. M., Scheinman P., Lantagne D. (2017). Handwashing and *E. bola* virus disease outbreaks: a randomized comparison of soap, hand sanitizer, and 0.05% chlorine solutions on the inactivation and removal of model organisms Phi6 and *E. coli* from hands and persistence in rinse water. *PloS one*.

[10] Mourad K. A., Hobro S. (2020). Developing chlorine-based antiseptic by electrolysis. *Science of The Total Environment*.

[11] Anagnostopoulos A. G., Rong A., Miller D. (2018). 0.01% hypochlorous acid as an alternative skin antiseptic: an in vitro comparison. *Dermatologic Surgery*.

[12] Sakarya S., Gunay N., Karakulak M., Ozturk B., Ertugrul B. (2014). Hypochlorous acid: an ideal wound care agent with powerful microbicidal, antibiofilm, and wound healing potency. *Wounds: A Compendium of Clinical Research and Practice*.

[13] Wang L., Bassiri M., Najafi R. (2007). Hypochlorous acid as a potential wound care agent: part I. stabilized hypochlorous acid: a component of the inorganic armamentarium of innate immunity. *Journal of Burns and Wounds*.

[14] Ciccia M., Chakrokh R., Molinazzi D., Zanni A., Farruggia P., Sandri F. (2018). Skin antisepsis with 0.05% sodium hypochlorite before central venous catheter insertion in neonates: a 2-year single-center experience. *American Journal of Infection Control*.

[15] Petrosyan E. A. (2019). Russian federation (RU).

[16] ASTM International (2010). *ASTM E1115: Standard Test Method for Evaluation of Surgical Hand Scrub Formulations*.

[17] Yoon C., Gong H. S., Park J. S., Seok H. S., Park J. W., Baek G. H. (2019). Two-layer wound sealing before surgical hand washing for surgeons with a minor cut injury on the hand. *Surgical Infections*.

[18] Macinga D. R., Beausoleil C. M., Campbell E. (2011). Quest for a realistic in vivoTest method for antimicrobial hand-rub agents: introduction of a low-volume hand contamination procedure. *Applied and Environmental Microbiology*.

[19] Rotter M., Sattar S., Dharan S., Allegranzi B., Mathai E., Pittet D. (2009). Methods to evaluate the microbicidal activities of hand-rub and hand-wash agents. *Journal of Hospital Infection*.

[20] ASTM International (2008). *E1054–08: Standard Test Methods for Evaluation of Inactivators of Antimicrobial Agents*.

[21] De Muth J. E. (2014). *Basic Statistics and Pharmaceutical Statistical Applications*.

[22] Olson L. K. M., Morse D. J., Duley C., Savell B. K. (2012). Prospective, randomized in vivo comparison of a dual-active waterless antiseptic versus two alcohol-only waterless antiseptics for surgical hand antisepsis. *American Journal of Infection Control*.

[23] Kashimatanaka M., Tsujimoto Y., Kawamoto K., Senda N., Ito K., Yamazaki M. (2003). Generation of free radicals and/or active oxygen by light or laser irradiation of hydrogen peroxide or sodium hypochlorite. *Journal of Endodontics*.

[24] Cui R. R., Deng L. Z., Shi W. B., Yang H. P., Sha G. H., Zhang C. H. (2011). Liquid-liquid reaction of hydrogen peroxide and sodium hypochlorite for the production of singlet oxygen in a centrifugal flow singlet oxygen generator. *Quantum Electronics*.

[25] Dahl T. A., Midden W. R., Hartman P. E. (1989). Comparison of killing of gram-negative and gram-positive bacteria by pure singlet oxygen. *Journal of Bacteriology*.

